# Evaluation of the thyroid characteristics of patients with growth hormone-secreting adenomas

**DOI:** 10.1186/s12902-019-0424-x

**Published:** 2019-09-02

**Authors:** Dianshuang Xu, Bolin Wu, Xiaoju Li, Yunjiu Cheng, Dubo Chen, Yuefeng Fang, Qiu Du, Zhiyong Chen, Xiaodong Wang

**Affiliations:** 1 Department of Neurosurgery, The First Affiliated Hospital, Jinan University, No. 613 Huangpu Avenue West, Tianhe District, Guangzhou 510632, Guangdong Province 510630 People’s Republic of China; 2 Department of Medical Ultrasound, First Affiliated Hospital, Sun Yat-Sen University, Guangzhou, 510632 China; 3 Department of cardiology, The First Affiliated Hospital, SunYat-sen University, Guangzhou, 510632 China; 4 Department of laboratory medicine, The first affiliated hospital, Sun Yat-sen university, Guangzhou, 510632 China; 5 Asset Management Division, Guangdong University of Foreign Studies, Guangzhou, 510420 China; 6 Department of Neurosurgery, The Affiliated Hospital of Yangzhou University, Yangzhou, 225012 China

**Keywords:** Thyroid disease, Growth hormone secreting adenomas, Thyroid nodules, Ultrasound

## Abstract

**Background:**

Acromegaly is highly associated with thyroid disorders. However, the clinical characteristics of thyroid nodules in individuals with acromegaly who present with thyroid diseases have not been completely elucidated.

**Methods:**

Overall, 134 consecutive participants with growth hormone (GH)-secreting adenoma (*n* = 67) and non-functioning (NF) pituitary adenoma (n = 67) were recruited from the outpatient and inpatient patient department of The First Affiliated Hospital, Jinan University from August 2015 to August 2017. Thyroid ultrasonography was performed using an ultrasound system. The cytopathological results of fine-needle aspiration biopsy were analyzed by a pathologist according to the Bethesda system. Twenty-one patients with GH-secreting adenoma and thyroid disease underwent transsphenoidal pituitary adenoma resection and were followed up for 1 year.

**Results:**

The prevalence of thyroid disease increased in the GH-secreting adenoma group compared with that in the NF pituitary adenoma group. The number of hypoechoic, isoechogenic, heterogeneous, and vascular thyroid nodules increased in patients with GH-secreting adenoma plus thyroid disease compared with that in patients with NF pituitary adenoma plus thyroid disease. Finally, we found significant decreases in the morphology of solid nodules and significant increases in the morphology of cystic nodules after surgery compared with those before surgery in the cured group. Moreover, the numbers of heterogeneous and vascular thyroid nodules decreased significantly after surgery compared with those before surgery in the cured group. However, the characteristics of the thyroid nodules did not change after surgery compared with those before surgery in the non-cured group.

**Conclusions:**

The numbers of hypoechoic, isoechoic, heterogeneous, and vascular thyroid nodules increased in patients with GH-secreting adenomas. In these patients, surgery resulted in significant changes from solid to cystic nodules and also reduced the numbers of heterogeneous and vascular thyroid nodules.

## Background

Pituitary adenoma is a chronic endocrine disease that is characterized by the excessive secretion of growth hormone (GH) and insulin-like growth factor I (IGF-I), which results in various physiological abnormities [1, 2]. Increasing evidence has shown that many individuals with acromegaly also present with various thyroid abnormities [3–5]. Golkowski et al. reported goiter in 85 (75.2%) of 113 patients with acromegaly [6]. In addition, Roqozinski et al. observed nodular thyroid disorders in 23 (67%) of 34 patients with acromegaly [5]. Moreover, Gasperi et al. reported that 202 (78.3%) of 258 patients with acromegaly and 28 (10.8%) of 258 patients with non-functioning prolactin-secreting pituitary adenomas were diagnosed with thyroid disorders [7]. Furthermore, the risk of thyroid cancer is high in patients with acromegaly [8, 9]. The results of these studies indicate that the thyroid is the most frequently affected organ in patients with acromegaly; hence, the correlation between acromegaly and thyroid disorders has become a topic of interest.

The development of thyroid nodules is caused by the abnormal proliferation of thyroid cells. The characteristics of thyroid nodules, including the number of nodules (single or multiple), morphology (solid, mixed, or cystic), position (right, left, or gorge), echogenicity (hypoechoic, isoechogenic, hyperechoic, or none), echotexture (homogeneous or heterogeneous), margin (regular or irregular), calcification (none, micro-calcification, or macro-calcification), and blood vessels (none, rare, or abundant), can be used as a basis for diagnosis [10]. However, the clinical characteristics of thyroid nodules in patients with acromegaly who were diagnosed with thyroid disease remain unclear.

This retrospective study analyzed the incidence of thyroid disease and the characteristics of thyroid nodules in patients with active growth hormone (GH)-secreting and non-functioning (NF) pituitary adenomas. We also analyzed the characteristics of thyroid nodules in patients with GH-secreting adenoma after surgery. This study was conducted to better understand the correlation between acromegaly and the characteristics of thyroid nodules.

## Methods

### Participants

All 134 consecutive participants with GH-secreting (*n* = 67) and NF pituitary (n = 67) adenomas were recruited from the outpatient and inpatient department of The First Affiliated Hospital, Jinan University between August 2015 and August 2017. After obtaining clinical parameters, such as age, sex, weight, basal metabolic rate, body mass index, heart rate, and blood pressure, the patients were assessed for the signs and symptoms of thyroid diseases. After measuring hormone levels and conducting ultrasonography, the patients were divided into two groups: those with pituitary adenoma who presented with a saddle area on computed tomography (CT) scan or magnetic resonance imaging (MRI), low serum IGF-1 levels, or pituitrin or prolactin levels < 100 ng/mL (NF pituitary adenoma group) and those with one of following symptoms: presence of a pituitary adenoma as characterized by a saddle area on CT scan or MRI, high serum IGF-1 levels, GH levels > 2.5 ng/mL, and low GH suppression (< 2 ng/mL) after the oral administration of 75 g of glucose (GH-secreting adenoma group). The exclusion criteria were as follows: 1) pregnant and lactating women, 2) those with psychiatric disorders, 3) those with pituitary disease diagnosed before acromegaly, 4) those who underwent thyroidectomy, 5) those who smoked or consumed alcohol, 6) those aged older than 70 years or younger than 20 years, and 7) those with other aggressive types of cancer. Prior to performing any procedure, each patient provided voluntary and written informed consent and written approval was obtained by the ethics committee of The First Affiliated Hospital, Jinan University.

### Endocrine examination and MRI

Venous blood was collected from all participants after they had fasted for 8–10 h. Levels of GH, IGF-1, and other pituitary endocrine hormones were measured using a chemiluminescent immunoassay system. Conventional and enhanced MRI scans of the pituitary gland were performed on all patients. The steps of the procedure were as described in our previous studies [11].

### Ultrasonography

Thyroid ultrasonography was performed by a practitioner using an iU22 xMatrix-DS Ultrasound system (PHILIPS, 795112) with a 3–12-MHz linear transducer. The thyroid volume was calculated using the volume formula for an ellipsoid model (π/6 × width × length × thickness). The sum of the volume of each thyroid lobe and isthmus was defined as the total thyroid volume [7]. Goiter was defined as a thyroid volume greater than 12.6 cm^3^ in women and 17.1 cm^3^ in men. After determining the thyroid volume, the patients were classified into the normal, diffuse goiter, and nodular goiter groups according to the thyroid morphology.

### Fine-needle aspiration biopsy (FNAB)

FNAB was performed by a practitioner on patients with nodule volume exceeding 1 cm^3^ or a node with two or more of diverse signals: 1) hypoechoic nodules, 2) hazy nodular border, 3) uneven internal echo, 4) abundant blood flow in the middle of the nodule, 5) multifocal strong echo, and 6) tophaceous calcification. The cytopathological results were analyzed by the same pathologist according to the Bethesda system [12].

### Treatment and follow-up

Forty-seven patients with GH-secreting adenoma and thyroid disease underwent relevant examinations and transsphenoidal pituitary adenoma resection, while three patients with thyroid cancer underwent thyroidectomy. All three patients with cancer and 23 other patients were lost to follow-up and were excluded from the study. After the 1-year follow-up, clinically relevant data from the remaining patients (*n* = 21) were collected.

### Statistical analysis

Statistical analyses were performed using IBM SPSS Statistics for Windows, version 19.0, and the results were expressed as medians (minimum-maximum). Pairwise comparisons were conducted using Wilcoxon rank-sum tests. f: Fisher’ exact test: 1) chi-square tests for *n* < 40 and 1 ≤ T < 5, 2) Yates’ chi-square test for *n* ≥ 40 and T ≥ 5, and 3) Fisher’s exact probability tests for n < 40 and T < 1. *P*-values < 0.05 were considered statistically significant.

## Results

**Fig. 1:**
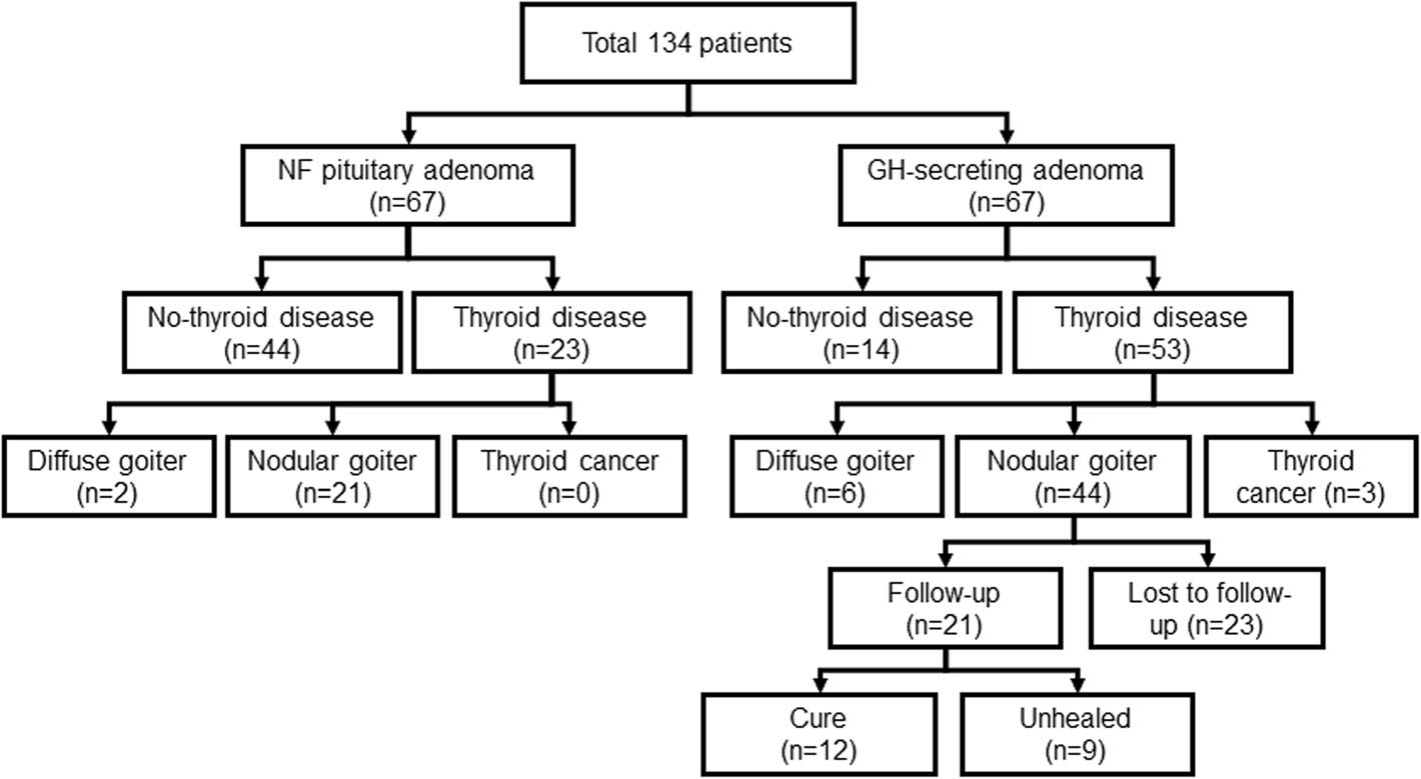
Diagram showing the number of included patients based on the inclusion and exclusion criteria

Figure 1 shows the number of patients included in the study based on the inclusion and exclusion criteria.

### High prevalence of thyroid disease in patients with GH-secreting adenomas

**Fig. 2:**
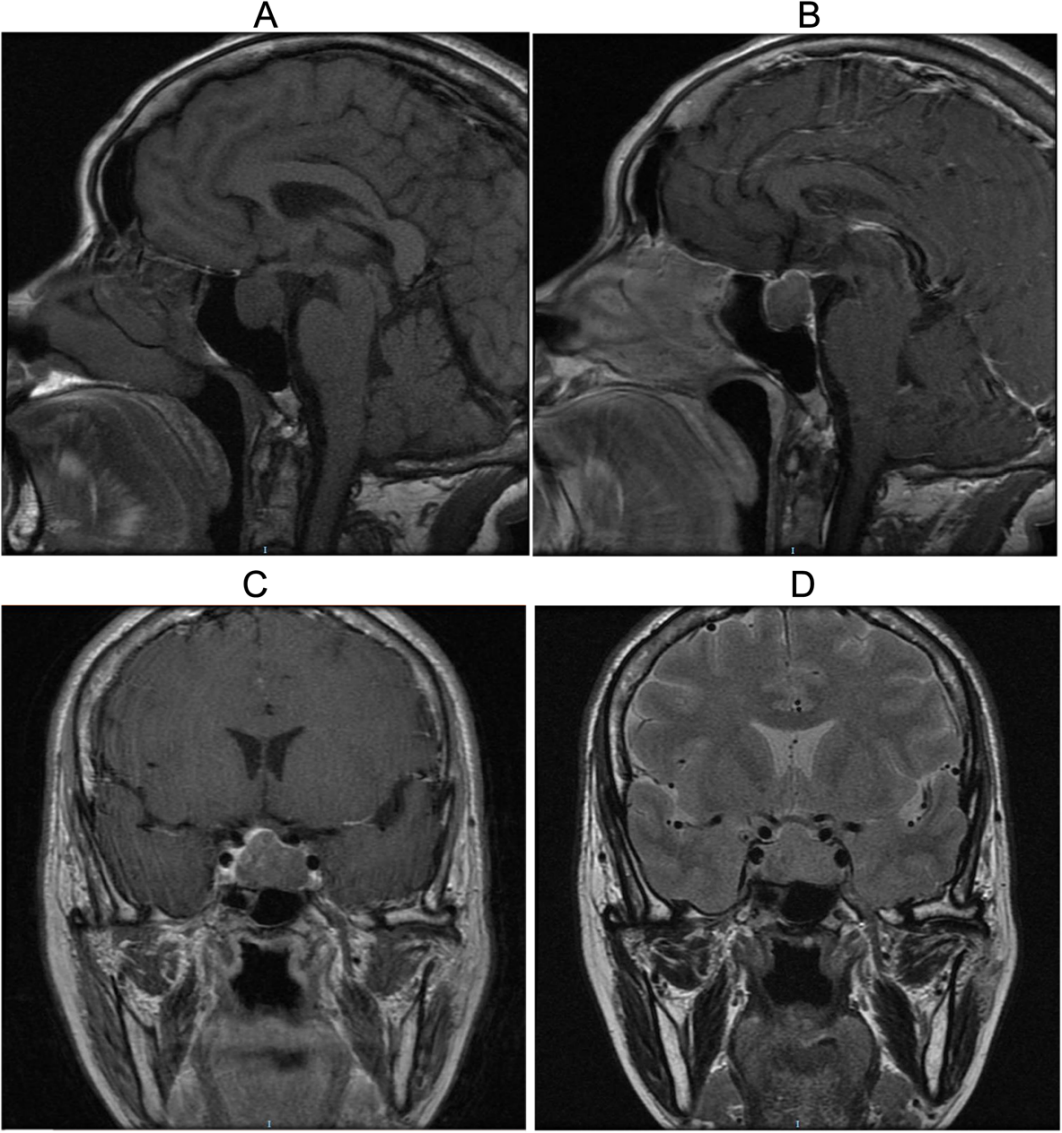
Representative image of a pituitary adenoma detected in magnetic resonance imaging. The sella is expanded within which a pituitary adenoma is visible. T1-weighted images showed uniform signals (**a**) and were slightly enhanced with contrast (**b** and **c**), whereas T2-weighted images had slightly higher signals (**d**)

**Fig. 3:**
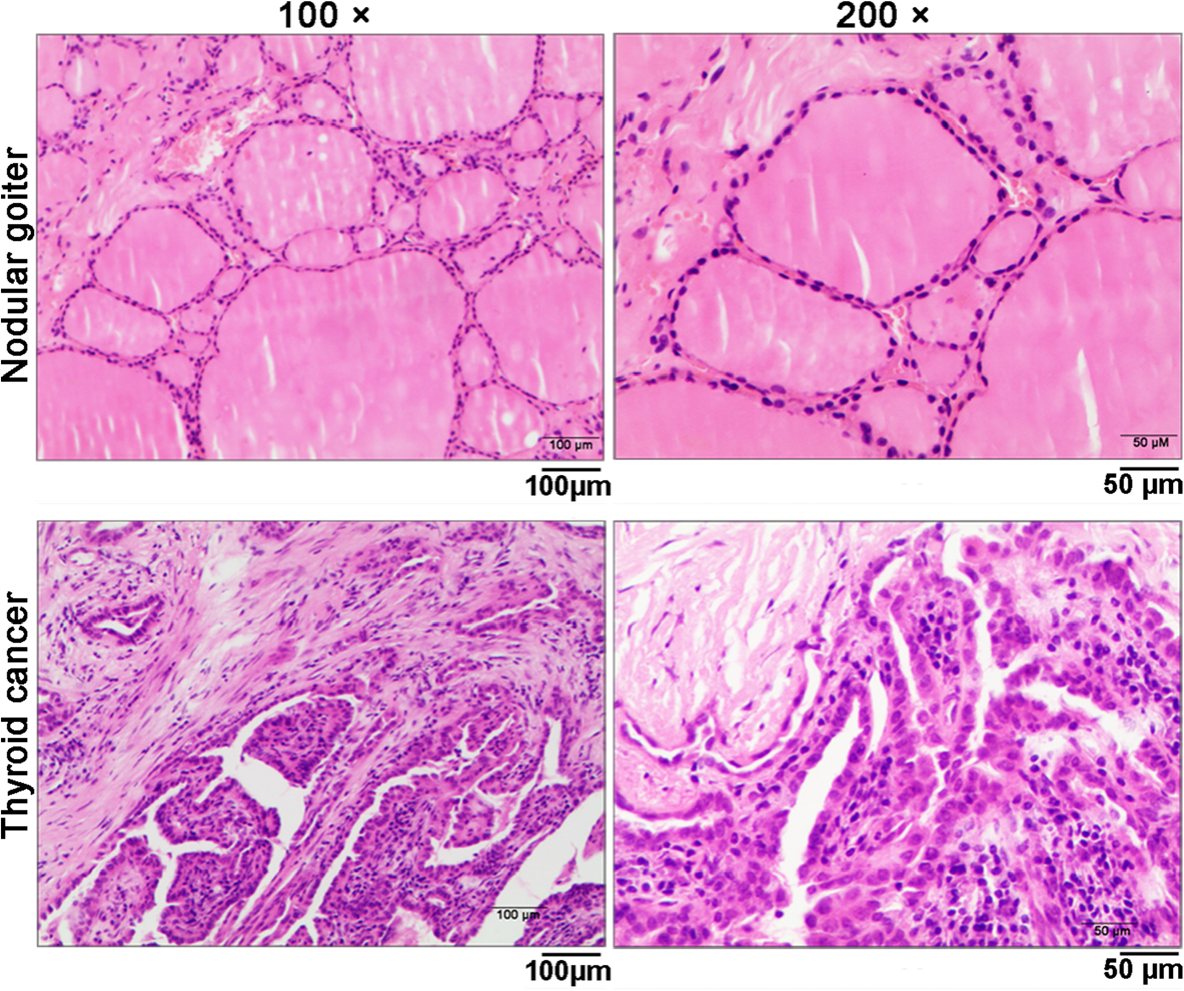
Representative images of nodular goiter and thyroid cancer detected by hematoxylin and eosin staining

**Fig. 4:**
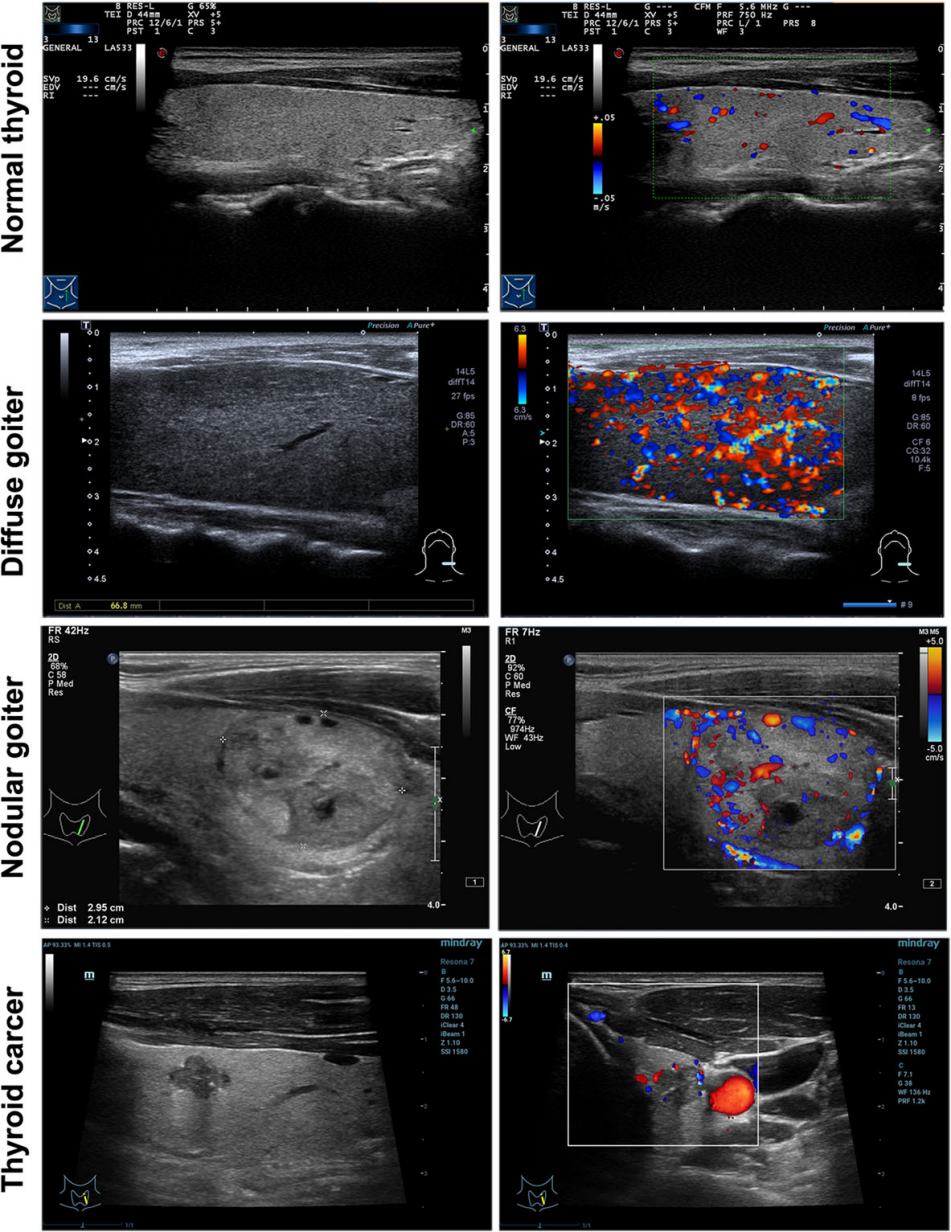
Representative images of nodular goiter and thyroid cancer detected by thyroid ultrasonography

**Fig. 5:**
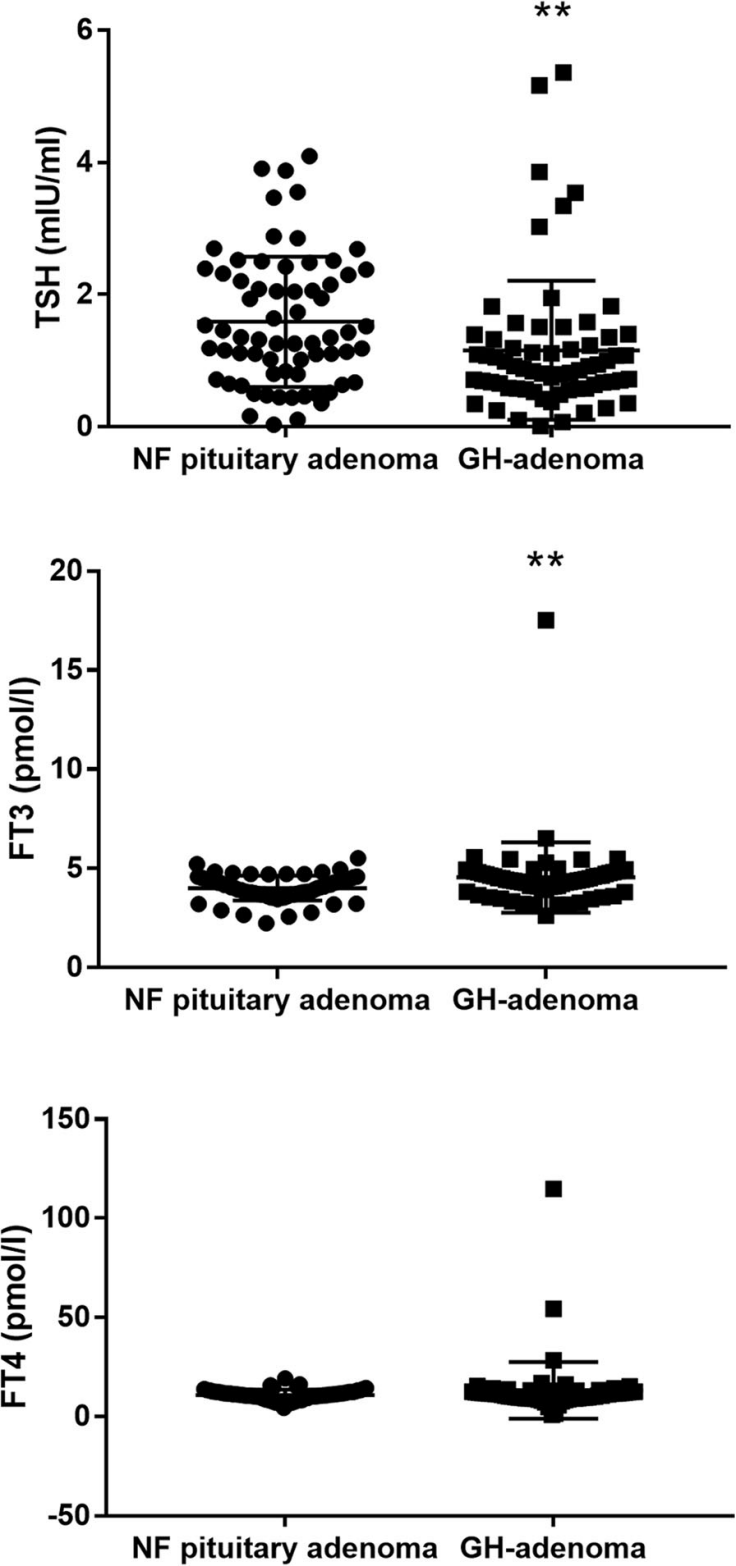
TSH, FT3, and FT4 were measured in GH-secreting adenoma and NF pituitary adenoma groups. Levels of TSH and FT3 were significantly increased while that of FT4 did not change significantly in the GH-secreting adenoma group compared with the NF pituitary adenoma group. **P < 0.01. GH: growth hormone; NF: non-functioning; TSH: thyroid-stimulating hormone; FT3: free triiodothyronine; FT4: free thyroxine

Figure 2 shows a representative pituitary adenoma in MRI showing an expanded sella with a pituitary adenoma inside. The histopathological morphology of thyroid disorders detected by hematoxylin and eosin staining is shown in Fig. 3. The pathological manifestations of nodular goiter were as follows: part of the follicular epithelium presented with columnar or papillary-like hyperplasia, formation of small follicles, part of the epithelium visible as old or atrophic, colloidal storage, interstitial fibrous tissue hyperplasia, and interval wrapping forming the different sizes of nodular lesions. The pathological manifestations of thyroid cancer were as follows: large numbers of nipple branches; vascular interstitial fibrosis at the center of the nipple and visible granules; nipple epithelium appearing as single or multi-layer cancer cells with varying degrees of differentiation; visible nuclear chromatin appearing as less, frosted glass or transparent; and lack of nucleoli. The morphological characteristics were analyzed by thyroid ultrasonography assay (Fig. 4). The characteristics of diffuse goiter, nodular goiter, and papillary thyroid cancer are shown in Table 1. The characteristics of diffuse goiter were a slightly thicker thyroid parenchyma on echo imaging, lack of lesions, and increased blood supply. The characteristics of nodular goiter included an irregular thyroid gland, uneven envelope, uneven internal echo, and normal blood supply. The thyroid gland presented with multiple nodules, mixed echoes, partial nodule liquefaction, partial nodules with coarse calcification, clear boundaries, and limited blood supply. The characteristics of papillary thyroid cancer were a slightly thicker thyroid parenchyma echo, low echogenicity, presence of many small calcified nodules, unclear border, and abundant peripheral blood supply. The incidence of thyroid disorders is shown in Table 2. The prevalence of thyroid disease was higher in the GH-secreting adenoma group than in the NF pituitary adenoma group (79.1% vs. 34.3%, *P* < 0.05). Among the 76 patients with thyroid disease, six of eight patients who presented with diffuse thyroid and 44 of the 65 patients with nodular goiter had GH-secreting adenomas. Notably, all three patients with thyroid carcinoma had GH-secreting adenomas. This result indicates the potential strong correlation between GH-secreting adenoma and aggressive thyroid disorders. Additionally, the TSH and FT3 levels were significantly increased, whereas the FT4 level had no significant change in the GH-secreting adenoma group compared with that in the NF pituitary adenoma group (Fig. 5).

**Table 1:** The characteristics of diffuse goiter, nodular goiter, and papillary thyroid cancer

Characteristics		Diffuse goiter	Nodular goiter	Papillary thyroid cancer
Single & multiple	Single	0	Y (6 patients)	Y (2 patients)
	Multiple	0	Y (38 patients)	Y (1 patient)
Total nodule		0	187	4
Morphology	Solid	/	Y	Y
	Mix	/	Y	N
	Cystic	/	Y	N
Echogenicity	Hypoechoic	/	Y	Y
	Isoechogenic	/	Y	N
	Hyperechoic	/	Y	Y
	None	/	Y	N
Echotexture	Homogeneous	/	Y	Y
	Heterogeneous	/	Y	Y
Margin	Regular	/	Y	Y
	Irregular	/	Y	Y
Calcification	None	/	Y	Y
	Micro	/	Y	Y
	Macro		Y	N
Blood vessel	None	/	Y	N
	Rare	/	Y	Y
	Rich	/	Y	Y

**Table 2:** Comparisons of thyroid disorders between the GH-secreting and NF pituitary adenoma groups

Variables	Total (*N* = 134)	NF (*n* = 67)	GH (*n* = 67)	*χ* ^*2*^	*P*
No thyroid disease	58	44	14	27.359^a^	< 0.001
Thyroid disease	76	23	53		
Diffuse goiter	8	2	6		
Nodular goiter	65	21	44		
Thyroid cancer	3	0	3		

*GH* Growth hormone secreting pituitary adenoma, *NF* Non-function pituitary adenomas

^a^Normal vs. thyroid disease

### Significant increase in the number of thyroid nodules in patients with GH-secreting adenoma

The characteristics of the thyroid nodules in patients with GH-secreting and NF pituitary adenomas are shown in Table 3. Among those with GH-secreting adenomas, the proportion of patients with multiple thyroid nodules was significantly higher than that of patients with single thyroid nodules. In addition, the proportion of patients with GH-secreting hormone adenomas who presented with multiple thyroid nodules (80.9%) was significantly higher than that of patients with NF pituitary adenomas (42.9%). The proportion of patients with hypoechoic, isoechoic, heterogeneous, and vascular lesions was significantly higher in the GH-secreting adenoma group than in the NF pituitary adenoma group.

**Table 3:** Comparisons of thyroid nodule characteristics between the GH-secreting and NF pituitary adenoma groups

Characteristics	Groups	NF (*n* = 21)	GH (*n* = 47)	*χ* ^*2*^	P
Single & multiple	Single	12 (57.1%)	9 (19.1%)	9.816	0.002*
	Multiple	9 (42.9%)	38 (80.9%)		
Total nodule		39	191		
Morphology*	Solid	20 (51.3%)	98 (51.3%)	0.013	0.994
	Mix	17 (43.6%)	84 (44.0%)		
	Cystic	2 (5.1%)	9 (4.7%)		
Location	Right	21 (53.9%)	95 (49.7%)	5.917	0.052
	Left	14 (35.9%)	91 (47.6%)		
	Isthmus	4 (10.3%)	5 (2.6%)		
Echogenicity	Hypoechoic	25 (64.1%)	146 (76.4%)	9.972	0.019*
	Isoechogenic	4 (10.3%)	27 (14.1%)		
	Hyperechoic	10 (25.6%)	16 (8.4%)		
	None	0 (0%)	2 (1.0%)		
Echotexture	Homogeneous	25 (64.1%)	82 (42.9%)	5.835	0.016*
	Heterogeneous	14 (35.9%)	109 (57.1%)		
Margin	Regular	25 (64.1%)	144 (75.4%)	2.118	0.146
	Irregular	14 (35.9%)	47 (24.6%)		
Calcification	None	37 (94.9%)	177 (92.7%)	1.464	0.481
	Micro	0 (0%)	6 (3.1%)		
	Macro	1 (5.1%)	8 (4.2%)		
Blood vessel	None	6 (15.4%)	4 (2.1%)	15.156	0.001*
	Rare	32 (82.1%)	169 (88.5%)		
	Rich	1 (2.6%)	18 (9.4%)		

Note: *P < 0.05

### Follow-up

Overall, 21 patients underwent transsphenoidal pituitary adenoma resection and were followed up for 1 year. Among them, 12 cases were cured, while nine cases were not cured. The morphological characteristics were analyzed by thyroid ultrasonography assay, the results of which are shown in Table 4. The number of thyroid nodules did not significantly change before and after surgery in the cured and non-cured groups. In addition, the number of patients with cystic nodules was significantly increased, while the number of solid nodules, heterogeneous nodules, and vascular thyroid nodules after surgery significantly decreased compared with those before surgery in the cured group. The characteristics of the thyroid nodules did not change significantly after surgery compared with those before surgery in the non-cured group.

**Table 4:** Thyroid nodule characteristics before and after surgery in the cured and non-cured groups

Characteristics		Cured (*n* = 12)		Non-cured (*n* = 9)	
		Before	After	Before	After
Single & multiple	Single	10 (83.3%)	10(83.3%)	8 (88.9%)	8 (88.9%)
	Multiple	2 (16.7%)	2 (16.7%)	1 (11.1%)	1 (11.1%)
Total nodule		41	40	46	63
Morphology*	Solid	16 (39.0%)	2 (5.0%)	20 (43.5%)	31 (49.2%)
	Mix	25 (61.0%)	10 (25.0%)	21 (45.7%)	25 (39.7%)
	Cystic	0 (0%)	28 (70.0%)	5 (10.9%)	7 (11.1%)
Location	Right	18 (43.9%)	18 (45.0%)	21 (45.7%)	26 (41.3%)
	Left	22 (53.7%)	21 (52.5%)	22 (47.8%)	31 (49.2%)
	Isthmus	1 (2.4%)	1 (2.5%)	3 (6.5%)	6 (9.5%)
Echogenicity	Hypoechoic	35 (85.4%)	35 (87.5%)	28 (60.9%)	38 (60.3%)
	Isoechogenic	2 (4.9%)	2 (5.0%)	11 (23.9%)	17 (27.0%)
	Hyperechoic	4 (9.8%)	3 (7.5%)	7 (15.2%)	8 (12.7%)
	None	0 (0%)	0 (0%)	0 (0%)	0 (0%)
Echotexture*	Homogeneous	16 (39.0%)	24 (60.0%)	19 (41.3%)	26 (41.3%)
	Heterogeneous	25 (61.0%)	16 (40.0%)	27 (58.7%)	37 (58.7%)
Margin	Regular	22 (53.7%)	28 (70.0%)	41 (89.1%)	50 (79.4%)
	Irregular	19 (46.3%)	12 (30.0%)	5 (10.9%)	13 (20.6%)
Calcification	None	36 (87.8%)	35 (87.5%)	42 (91.3%)	57 (90.5%)
	Micro	1 (2.4%)	1 (2.5%)	0 (0%)	2 (3.2%)
	Macro	4 (9.8%)	4 (10.0%)	4 (8.7%)	4 (6.3%)
Blood vessel*	None	3 (7.3%)	32 (80.0%)	0 (0%)	0 (0%)
	Rare	36 (87.8%)	8 (20.0%)	46 (100.0%)	58 (92.1%)
	Rich	2 (4.9%)	0 (0%)	0 (0%)	5 (7.9%)

Note: *P < 0.05, before surgery vs. after surgery, in the cured group

## Discussion

This study assessed the characteristics of thyroid nodules before and after surgery in patients with acromegaly who presented with GH-secreting adenoma. We observed an increased prevalence of thyroid disease in the GH-secreting adenoma group compared with that in the NF pituitary adenoma group. The numbers of hypoechoic, isoechogenic, heterogeneous, and vascular thyroid nodules increased in patients with GH-secreting adenoma plus thyroid disease compared with those in patients with NF pituitary adenoma plus thyroid disease. Finally, the morphology of solid nodules changed significantly to cystic nodules after surgery compared with that before surgery in the cured group. The numbers of heterogeneous and vascular thyroid nodules decreased significantly after surgery compared with those before surgery in the cured group. However, no significant changes were observed in the non-cured group.

The thyroid is the most frequently affected organ in patients with acromegaly, with more than 50% of patients with acromegaly presenting with thyroid disorders [13]. The development of thyroid diseases in patients with acromegaly is correlated to the excessive secretion of IGF-1 and growth hormones [14, 15]. Our previous study found significantly higher secretion of IGF-1 and growth hormones in the GH-secreting adenoma group than in the NF pituitary adenoma group [11]. We also found significantly increased TSH and FT3 levels and no significant change in FT4 level in the GH-secreting adenoma group compared with those in the NF pituitary adenoma group, thus suggesting that GH-secreting adenomas were correlated with thyroid dysfunction. Miyakawa noted that GH and IGF-1 levels were correlated to thyroid volume [16]. Moreover, patients with normal serum IGF-1 levels had smaller thyroid glands than those in patients with active acromegaly [17, 18]. Mechanistically, IGF-I could regulate cellular proliferation by stimulating various signaling pathways such as the Ras/Raf/MEK/ERK pathways [19]. IGF-I level was also associated with the development of thyroid cancers by inducing anti-apoptosis and proliferation of thyroid cancer cells [20, 21]. This study evaluated 67 patients with acromegaly, 79.1% of whom presented with thyroid abnormities, including diffuse and nodular goiter and thyroid carcinoma. Moreover, thyroid ultrasonography showed the highest number of patients in the group with multinodular goiter, consistent with a previous study showing that multinodular goiter is the most prevalent thyroid disorder in patients with acromegaly [22]. Another follow-up study in the US reported thyroid disorders in 69.5% of patients with acromegaly, including 47.8% with nodules. Further examinations performed on 64 patients with thyroid abnormities showed that 48.4% had multinodular goiter and 7.8% developed differentiated thyroid cancer [23]. Notably, the patients diagnosed with papillary thyroid carcinoma were all women and all thyroid cancers were papillary cancer. This result is consistent with that of previous studies showing that papillary cancer was the most common carcinoma among differentiated thyroid cancers [7, 24, 25]. In addition, previous studies have reported that thyroid disorders are more prevalent in women than in men and that papillary cancer is more commonly observed in woman than in men with acromegaly [7, 26].

The clinical features of thyroid nodules are significantly correlated to their status as benign or malignant. Hypoechoic, heterogeneous, and vascular nodules; those with irregular margin; and those with microcalcifications are closely correlated to thyroid nodules with malignant tendencies [27]. In this study, the proportion of patients with multiple thyroid nodules in the GH group (80.9%) was significantly higher than that in the NF group (42.9%). In addition, the number of hypoechoic, isoechogenic, heterogeneous, and vascular nodules increased in patients with GH-secreting adenoma. These results indicated that the thyroid nodules in patients with GH are more likely to be malignant. The clinical characteristics of thyroid goiter provide valuable information for the treatment and prognosis of thyroid abnormities in patients with acromegaly and the identification of benign and malignant thyroid nodules.

Transsphenoidal pituitary adenoma resection is a common surgery used to treat patients with acromegaly. Our previous study found that successful surgery in patients with acromegaly could significantly reduce GH/IGF-1 expression [11], which was associated with disease development [28]. Patients with acromegaly were also successfully treated with a somatostatin analog, which reduced the thyroid nodule volume [29]. Successful surgery in patients with acromegaly could lead to significant changes from solid to cystic nodules and reduce the numbers of heterogeneous and vascular nodules. However, no significant change was observed in the non-cured group between after surgery and before surgery. These results suggest that a successful transsphenoidal pituitary adenoma resection results in decreased GH/IGF-1 expression, thereby reducing the number of malignant lesions on the thyroid nodules. These changes in thyroid nodule characteristics can be used as a diagnostic tool to assess the efficacy of transsphenoidal pituitary adenoma resection.

The most significant limitations of our study were the small sample size and short follow-up time.

## Conclusions

The number of hypoechoic, isoechogenic, heterogeneous, and vascular thyroid nodules was higher in patients with GH-secreting adenoma. Successful treatment with surgery in patients with GH-secreting adenoma resulted in significant changes from solid to cystic nodules and reduced numbers of heterogeneous and vascular thyroid nodules. The reason for these changes may be due to decreased levels of growth hormone after resection of pituitary growth hormone adenomas, resulting in decreased thyroid cell proliferation and increased apoptosis. At the same time, the blood supply is reduced after reduction of thyroid cells; thus, the solid nodules are transformed into cystic nodules. However, these reasons require confirmation in further studies.

## Data Availability

The datasets used and/or analysed during the current study are available from the corresponding author on reasonable request.

## Abbreviations

GH: Growth hormone
IGF-I: Insulin-like growth factor I
NF: Non-functioning
